# Sensitive Lateral Flow Immunoassay of Beta-Agonist Mabuterol in Meat Samples Using Au@CeO_2_ Nanoparticles as a Catalytic Signal-Enhancing Label

**DOI:** 10.3390/foods15203732

**Published:** 2026-10-20

**Authors:** Elena A. Zvereva, Vasily G. Panferov, Polina S. Lukina, Olga D. Hendrickson, Xinxin Xu, Chuanlai Xu, Boris B. Dzantiev, Anatoly V. Zherdev

**Affiliations:** 1 A.N. Bach Institute of Biochemistry, Research Center of Biotechnology of the Russian Academy of Sciences, Leninsky Prospect 33, 119071 Moscow, Russia; zverevaea@yandex.ru (E.A.Z.); vaspanferov@gmail.com (V.G.P.); polinalukina2@gmail.com (P.S.L.); odhendrick@gmail.com (O.D.H.); dzantiev@inbi.ras.ru (B.B.D.); 2 State Key Lab of Food Science and Technology, School of Food Science and Technology, Jiangnan University, Wuxi 214122, China; xxx89826@163.com (X.X.); xcl@jiangnan.edu.cn (C.X.)

**Keywords:** mabuterol, beta-agonist, lateral flow immunoassay, Au@CeO_2_ nanoparticles, meat products, food control

## Abstract

The use of beta-adrenomimetics (beta-agonists) to enhance the productivity of farm animals can lead to their unintended ingestion by humans through contaminated food products. To ensure the effective detection and prevention of these risks—which arise even at low contaminant concentrations—a highly sensitive lateral flow immunoassay (LFIA) was developed for the detection of the beta-agonist mabuterol (MAB). We have comparatively evaluated two nanoparticle labels: conventional gold nanoparticles (AuNPs) and bimetallic core@shell Au@CeO_2_ nanoparticles (Au@CeO_2_ NPs). The assay protocols were systematically optimized, including immunoreagent concentrations, reaction media composition, and assay time. Exploiting the peroxidase-mimetic activity of Au@CeO_2_ NPs, a catalytic amplification strategy was integrated with the LFIA protocol. This was achieved by treating the test strip with a 3,3′,5,5′-tetramethylbenzidine-based substrate. Compared with the traditional AuNPs-based LFIA, Au@CeO_2_ NPs provided a 2.6-fold decrease in the instrumental limit of detection (0.6 ng/mL) in the one-step catalytic format and a 6-fold decrease (0.26 ng/mL) in the two-step catalytic format. The total assay time was 18 min. An assessment of matrix effects using beef, pork, and chicken meat extracts yielded detection rates ranging from 80% to 139%, indicating potential applicability of the developed method. Furthermore, the developed assay format, utilizing the catalytic properties of Au@CeO_2_ NPs, provides a promising platform for sensitive detection of other low-molecular-weight toxic contaminants in food safety monitoring.

## 1. Introduction

The global expansion of food production demands intensified agricultural methodologies. This intensification is frequently facilitated by the application of productivity-enhancing substances encompassing pesticides for weed and pest management, antibiotics for disease control in livestock and poultry, and growth promoters to increase yield [1]. The widespread use of these low-molecular-weight compounds results in their migration through production chains and their eventual presence in food products.

Mabuterol ((RS)-1-[4-amino-3-chloro-5-(trifluoromethyl)phenyl]-2-(tert-butylamino)ethanol) (MAB) is a long-acting β2-adrenoceptor agonist and bronchodilator. Within livestock production, beta-agonists such as MAB are employed to improve feed conversion efficiency, promote water retention, diminish fat deposition, and increase lean muscle mass [2,3]. They function by stimulating β-adrenergic receptors on cell membranes, acting as nutrient repartitioning agents that direct nutrients toward muscle growth and reduce fat accumulation [4]. Their principal mechanisms of action include the induction of protein synthesis and muscle hypertrophy via the inhibition of proteolysis, coupled with the promotion of lipolysis in adipose tissue.

Residues of beta-agonists present significant hazards to human health, adversely affecting the central nervous system and contributing to the pathogenesis of cardiovascular diseases. Due to these risks, their employment as growth promoters is strictly prohibited in a majority of countries [2]. Consequently, the development of rapid and highly sensitive detection methods for these compounds, particularly MAB, remains an important analytical challenge. Ensuring food safety requires highly sensitive methods for toxicant detection that integrate initial screening and subsequent confirmation. Various chromatographic techniques enable the identification and quantification of components in complex mixtures [5,6,7]. Thus, Pieragostini et al. demonstrated the feasibility of qualitatively detecting 21 beta-agonists, including mabuterol, at a level of 0.05 µg/kg [6]. However, these techniques require specialized equipment and qualified personnel and are thus focused on centralized services for definitive confirmatory analysis. For non-laborious screening of numerous samples, immunoanalytical methods relying on highly specific antigen–antibody interactions are well-suited. Their two main classes, supported by large-scale manufacturing technologies, are enzyme-linked immunosorbent assay (ELISA) and lateral flow immunoassay (LFIA). An example of ELISA realization is the work by Paleologo Oriundi et al., which reached a detection limit for MAB in buffer of 0.3 ng/mL [8]. But ELISA application requires laboratory equipment for microplate manipulations and the following photometric measurements in microwells. This limits ELISA use in initial food-safety screening and drives interest in rapid, non-laboratory testing, even if it is less sensitive (but better suited to practical monitoring demands). The advantages of LFIA are connected with its adaptation for out-of-laboratory implementation, high speed, and procedural simplicity [9,10,11].

Achieving lower limits of detection (LOD) for lateral flow immunoassays constitutes a critical analytical frontier. This imperative is accentuated in the analysis of solid matrices, where requisite extraction and dilution procedures during sample preparation can drastically attenuate assay sensitivity within complex, real-world samples. The inherent sensitivity constraints of LFIA systems utilizing the most conventional and robust label—gold nanoparticles (AuNPs)—have initiated active exploration of alternative signaling strategies [12]. A promising avenue for this purpose involves the deployment of multi-component noble metal nanoparticles endowed with nanozyme activity [13,14]. In such advanced LFIAs, following the completion of the immunoreaction, a specific substrate for the nanozyme is introduced onto the test strip. The ensuing catalytic reaction generates colored products, resulting in a substantial growth of the signal intensity. This enhancement directly translates to increased assay sensitivity, permitting the use of reduced concentrations of specific reagents and, consequently, enabling detection of lower concentrations of the target analyte in competitive assay formats.

In this study, bimetallic Au@CeO_2_ nanoparticles were employed to enhance assay detectability. The Ce(III)/Ce(IV) redox couple is a well-established source of peroxidase-like activity in CeO_2_-based nanozymes, with the relationship between the Ce(III)/Ce(IV) ratio and catalytic activity reported in numerous studies [15,16,17]. Au@CeO_2_ NPs were first described by [18] as a functional multienzyme nanozyme. The authors proposed that the close values of the redox potentials of Au+/Au and Ce(III)/Ce(IV) facilitate the formation of a redox couple, contributing to the multienzyme activity of Au@CeO_2_ NPs. Kinetic parameters demonstrated that the peroxidase-like activity of this nanozyme is maximal at acidic pH and is comparable to that of the natural enzyme, horseradish peroxidase (HRP). The application of such Au@CeO_2_ NPs in LFIA remains limited. Wang et al. reported sandwich LFIA using alloy Au-CeO_2_ NPs for detecting heart-type fatty acid-binding protein in serum from patients with acute myocardial infarction [19].

The present study aimed to develop an LFIA for the detection of the beta-agonist MAB, utilizing both conventional AuNPs and Au@CeO_2_ NPs as labels. The research encompassed the synthesis and characterization of analytical reagents, the determination of optimal conditions for the Au@CeO_2_ NP-based LFIA with signal amplification, and an evaluation of the proposed assay’s applicability for testing meat samples.

## 2. Materials and Methods

### 2.1. Chemicals and Materials

Mabuterol (MAB), clenbuterol, fenoterol, orciprenaline, ractopamine, salbutamol, terbutaline, chloroauric acid, N-hydroxysuccinimide (NHS), 1-ethyl-3-(3′-dimethylaminopropyl)carbodiimide (EDC), bovine serum albumin (BSA), keyhole limpet hemocyanin (KLH), sodium citrate, sodium azide, Triton X-100, 3,3′,5,5′-tetramethylbenzidine (TMB) and sucrose were purchased from Sigma Chemicals (St. Louis, MO, USA). HEPES (4-(2-hydroxyethyl)-1-piperazineethanesulfonic acid) was obtained from Suzhou Yacoo Science (Suzhou, China). MES 1-hydrate for buffer solutions was obtained from Applichem (Darmstadt, Germany). Polyclonal goat antibodies against mouse IgG (GAMI) and polyclonal donkey antibodies against goat IgG (DAGI) were from Arista Biologicals (Allentown, PA, USA). Horseradish peroxidase-conjugated GAMI (GAMI–HRP) was purchased from Jackson ImmunoResearch (Cambridgeshire, UK). A one-component HRP substrate solution based on TMB was obtained from Immunotech (Moscow, Russia). All other solvents and chemicals, of analytical grade or higher purity, were purchased from Khimmed (Moscow, Russia). Costar 9018 96-well transparent polystyrene microplates (Corning Costar, Tewksbury, MA, USA) were used for ELISA.

### 2.2. Obtaining Monoclonal Antibodies (MonoAb) Against MAB

The production of monoclonal antibodies against MAB was based on the common hybridoma technology [20] and presented in our earlier publication [21,22]. MAB was diazotized through its aromatic amino group and conjugated with KLH to obtain the immunogen used. The resulting monoclonal antibodies were secreted by the selected hybridoma cell line and subsequently isolated using affinity chromatography on a column containing the immunoglobulin-binding streptococcal protein G. All animal procedures reported in study [21] were conducted in accordance with the “Regulation for the Administration of Affairs Concerning Experimental Animals” of China and received approval from the Animal Ethics Committee of Jiangnan University.

### 2.3. ELISA of MAB

Coating antigen for ELISA and LFIA (see Section 2.7) was synthesized and described in our earlier work [21,22] by salbutamol derivatization using the succinic anhydride method and the following conjugation with BSA. Coating antigen (0.5 μg/mL, 100 μL) in 50 mM K-phosphate buffer with 0.1 M NaCl, pH 7.4 (PBS) was immobilized in the wells of a plate overnight at 4 °C. Following coating, the wells were washed four times with PBS supplemented with 0.05% Triton X-100 (PBST). A 50 μL aliquot of MAB prepared in PBST, at concentrations between 100 ng/mL and 0.5 pg/mL, was subsequently added to the wells together with 50 μL of specific MonoAb (2.0 ng/mL in PBST). The plates were incubated for 1 h at 37 °C. After another washing step with PBST, 100 μL of GAMI–HRP diluted 1:3000 in PBST was introduced into each well, followed by incubation for 1 h at 37 °C. The enzymatic activity of the bound HRP was measured using 100 μL of a TMB-based substrate. The enzymatic reaction was terminated with 50 μL of 1 M sulfuric acid, and the absorbance of the resulting reaction product was measured at 450 nm. Optical density (OD) measurements were performed using a Zenyth 3100 microplate photometer (Anthos Labtec Instruments, Salzburg, Austria).

### 2.4. Preparation and Characterization of AuNPs

AuNPs were prepared following the protocol originally reported by Frens [23]. To obtain the nanoparticles, 1.0 mL of 1% (*w*/*v*) chloroauric acid (HAuCl_4_) was mixed with 97.5 mL of deionized water. The resulting solution was brought to a boil, after which 1.5 mL of 1% (*w*/*v*) trisodium citrate was introduced while the mixture was continuously stirred. Boiling was maintained for a further 25 min. The suspension was subsequently allowed to cool and stored at 4–6 °C. For the preparation of AuNPs having an average particle diameter of 16.5 nm, the same synthesis procedure was carried out using an increased volume of 1% sodium citrate solution (3 mL).

The particle size and size distribution of the resulting AuNPs were examined by transmission electron microscopy (TEM). Imaging was performed with a JEM-100C microscope (JEOL, Tokyo, Japan) operated at an accelerating voltage of 80 kV, using magnifications between 66,000× and 100,000× [24]. For TEM analysis, aliquots of the AuNP suspension were deposited onto 300-mesh copper grids coated with Formvar. The resulting TEM images were subsequently processed and analyzed using Image Tool software, version 3.0 (University of Texas Health Science Center at San Antonio, TX, USA).

### 2.5. Preparation and Characterization of Au@CeO_2_ NPs

The Au@CeO_2_ nanoparticles were obtained using a modified overgrowth procedure adapted from the method reported by Piella et al. [25]. The as-synthesized AuNPs (19 mL) with an average diameter of 16.5 nm were then mixed with 1 mL of freshly prepared 100 mM sodium citrate. The pH was subsequently brought to 10.0 by addition of saturated Na_2_CO_3_ solution. The mixture was heated in a double-neck flask equipped with an Allihn condenser. Upon boiling (in the original protocol at 70 °C), 150 µL of 30 mM Ce(NO_3_)_3_ × 6H_2_O was rapidly injected, and the colloidal solution was maintained under reflux for 4 h (in the original protocol for 5 h). The resulting Au@CeO_2_ NPs were kept in glassware at 4 °C.

Au@CeO_2_ NPs (1 mL) were concentrated by centrifugation (12,000× *g*, 15 min). The obtained pellet was resuspended in 200 µL of Milli-Q water. An aliquot of the concentrated colloidal solution was incubated on an ultra-thin carbon-coated copper grid (Ted Pella Inc., Redding, CA, USA, #01824) at 37 °C for 20 min. The samples were analyzed using a JEM-F200 S/TEM transmission electron microscope (JEOL, Tokyo, Japan) equipped with energy-dispersive X-ray spectroscopy (EDX) and electron energy loss spectroscopy (EELS) modules. Statistical analysis and EDX and EELS data processing were performed using DigitalMicrograph, version 3.6 from Gatan Inc. (Pleasanton, CA, USA).

### 2.6. Synthesis of GAMI–AuNPs and GAMI–Au@CeO_2_ NPs Conjugates

Prior to conjugation, GAMI was dialyzed against 10 mM Tris-HCl buffer, pH 8.5. The AuNP and Au@CeO_2_ NP solutions (D_520_ = 1.0) were subsequently adjusted to pH 8.5 using 0.2 M K_2_CO_3_. GAMI was then introduced at final concentrations of 6 μg/mL for the AuNP dispersion [24] and 20 μg/mL for the Au@CeO_2_ NP dispersion. The resulting mixtures were stirred at room temperature for 45 min to facilitate antibody attachment. BSA was subsequently added to reach a final concentration of 0.25%, and stirring was continued for an additional 15 min. GAMI–AuNPs and GAMI–Au@CeO_2_ NPs conjugates were separated from non-interacted antibodies by centrifugation at 10,000× *g* for 15 min at 4 °C.

Following removal of the supernatant, the pellets were resuspended in 10 mM Tris–HCl buffer (pH 8.5) supplemented with 1% BSA, 1% sucrose, and 0.05% sodium azide (all concentrations expressed as *w*/*v*) and stored at 4 °C. During storage for a minimum of two months, neither visible precipitate formation nor substantial alterations in the spectral characteristics of the conjugates were detected. The hydrodynamic size of the prepared NP conjugates was determined by dynamic light scattering (DLS) using a Zetasizer Nano ZS 90 instrument (Malvern Panalytical, Malvern, UK).

### 2.7. Production of Immunochromatographic Test Strips

The immunochromatographic strips were prepared using a CNPC-12 working nitrocellulose membrane (Advanced Microdevices, Ambala Cantt, India). Reagent deposition onto the membrane was performed with an IsoFlow automatic dispenser (Imagene Technology, Hanover, NH, USA). For the formation of the control line (CL), DAGI solution in PBS at a concentration of 0.25 mg/mL was applied, whereas the test line (TL) was generated using the coating antigen at a concentration of 0.5 mg/mL in PBS. The CL and TL were positioned 4 mm apart, and both reagents were dispensed at a rate of 0.1 μL per 1 mm of membrane length. After reagent application, the membranes were allowed to dry at room temperature (RT) for a minimum of 24 h. The individual membrane components were then assembled into a multimembrane composite, from which test strips with a width of 3.0 mm were cut using a guillotine cutter (KinBio, Shanghai, China). Each resulting strip had a total length of 5 cm. The finished test strips were placed in sealed bags and stored at RT. Under these conditions, the strips retained their functional properties for at least two months. No statistically significant differences in signal level and LOD were found.

### 2.8. Sample Preparation

Raw meat samples were purchased from specialized organic food retail stores. The absence of veterinary drugs (antibiotics and growth factors) was tested and confirmed by the manufacturers, which allowed these products to be used in the study as negative matrices. Pretreatment of meat samples was performed using three different extraction procedures:

1. 1 g of homogenized meat was transferred into a 10 mL plastic tube and mixed with 2 mL of PBST. The suspension was stirred for 5 min and subsequently centrifuged at 10,000× *g* for 5 min, and the supernatant was collected for LFIA analysis [26].
2. 1 g of homogenized meat was weighed, mixed with 2 mL of PBS in a 10 mL plastic tube, and sonicated for 10 min. The resulting suspension was centrifuged at 10,000× *g* for 10 min. The supernatant was passed through a 0.22 μm membrane filter, diluted with PBS supplemented with 0.1% Triton X-100, and then used for LFIA analysis [27].
3. 2 g of homogenized meat was weighed and mixed with 6 mL of acetonitrile, vortexed vigorously for 10 s, shaken upside down for 15 min, and centrifuged for 10 min at 3000× *g* at RT. 4 mL of supernatant was transferred into a new glass vial and evaporated to complete dryness under a gentle stream of air or nitrogen at 60 °C. The resulting dry residue was reconstituted in 1 mL of wash buffer [28].

The resulting extracts were collected, divided into aliquots, and stored at −18 °C.

### 2.9. LFIA of MAB

MAB standard solutions were prepared at concentrations between 0.01 and 10 ng/mL using either PBST or the corresponding sample extracts as the diluent.

For the non-catalytic LFIA format employing GAMI–AuNPs, 50 µL of the sample was combined with 1 µL of the GAMI–AuNP conjugate (OD_520_ = 10.0) and 1 µL of MonoAb (3.1 µg/mL). The test strip was subsequently immersed in the prepared mixture and allowed to develop for 10 min.

The enhanced one-step LFIA procedure was carried out using GAMI–Au@CeO_2_ NPs. In this format, 50 µL of the sample was mixed with 1 µL of the GAMI–Au@CeO_2_ NP conjugate (OD_520_ = 12.0) and 1 µL of MonoAb (1.55 µg/mL). The test strip was immersed in the mixture for 10 min. Following this incubation, the strip was removed and positioned horizontally. A 5 µL aliquot of the HRP substrate (0.1 M sodium citrate buffer, pH 4.0, containing 0.2 mg/mL TMB, 60 mg/mL H_2_O_2_ and 1 mg/mL dextran sulfate) was then deposited onto the membrane region located between the TL and CL and allowed to react for 3 min.

For the enhanced two-step LFIA format based on GAMI–Au@CeO_2_ NPs, 50 µL of the sample was initially mixed with 1 µL of MonoAb (3.1 µg/mL). The test strip was vertically immersed in this mixture and incubated for 5 min. The strip was then transferred to 50 µL of PBST containing 1 µL of GAMI–Au@CeO_2_ NP conjugate (OD_520_ = 12.0), followed by a further 10 min incubation. Staining was performed similarly to the one-step analysis with GAMI–Au@CeO_2_ NPs.

All LFIA measurements were performed in triplicate using three test strips from a single batch on the same day to enable statistical evaluation. Following completion of the assays, the test strips were digitized at a resolution of 600 dpi using a Canon 9000F Mark II scanner (Canon, Tokyo, Japan). The resulting images were processed and quantitatively evaluated with TotalLab software, version 3.00 (TotalLab, Newcastle Upon Tyne, UK).

### 2.10. Analytical Performance of ELISA and LFIA

The dependences of optical density (OD) in ELISA and color intensity of TL in LFIA on MAB concentration were fitted in Origin 9.0 software (OriginLab Corp., Northampton, MA, USA) using a four-parameter sigmoidal model:

*y* = (*a* − *b*)/[1 + (*x*/*c*)*^d^*] + *b*,

where *a* denotes the upper asymptotic signal and *b* represents the lower asymptotic signal. The parameter *c*, corresponding to the IC_50_ value, represents the MAB concentration required to produce a 50% decrease in the signal, whereas *d* describes the slope of the curve at *c*.

The effective working interval of the assays was established from the MAB concentrations associated with 20% and 80% inhibition of the analytical signal. For instrumental measurements, LOD was assigned to the MAB concentration producing 10% signal inhibition [29]. In the case of LFIA, the visual LOD, or cutoff value, was defined as the lowest MAB concentration at which the TL became completely invisible to the naked eye.

### 2.11. Study of LFIA Cross-Reactivity and Assessment of Matrix Effects

Cross-reactivity (CR) was determined using the following formula:

CR = *IC*_50*MAB*_/*IC*_50*analog*_ × 100%

where IC_50_ denotes the concentration of an analyte required to inhibit 50% of the measured analytical signal.

Cross-reactivity was studied using other beta-agonists (salbutamol, ractopamine, orciprenaline, terbutaline, fenoterol) and veterinary drugs from other classes (ampicillin, chloramphenicol, streptomycin, tetracycline). The detection rate (DR, %) for spiked samples testing was calculated as follows:

DR = *x_exp_*/*x_theor_* × 100,

where *x_theor_* is the concentration of MAB added to the sample, and *x_exp_* is the concentration of MAB determined in the sample analysis using the calibration curve.

## 3. Results and Discussion

### 3.1. Preparation and Checking of Key Immunoreagents

The monoclonal antibodies (MonoAb) against MAB were characterized by ELISA, demonstrating the LOD value of 0.1 ng/mL and a working range of detectable concentrations of 0.18–1.05 ng/mL (Figure S1).

**Figure 1:**
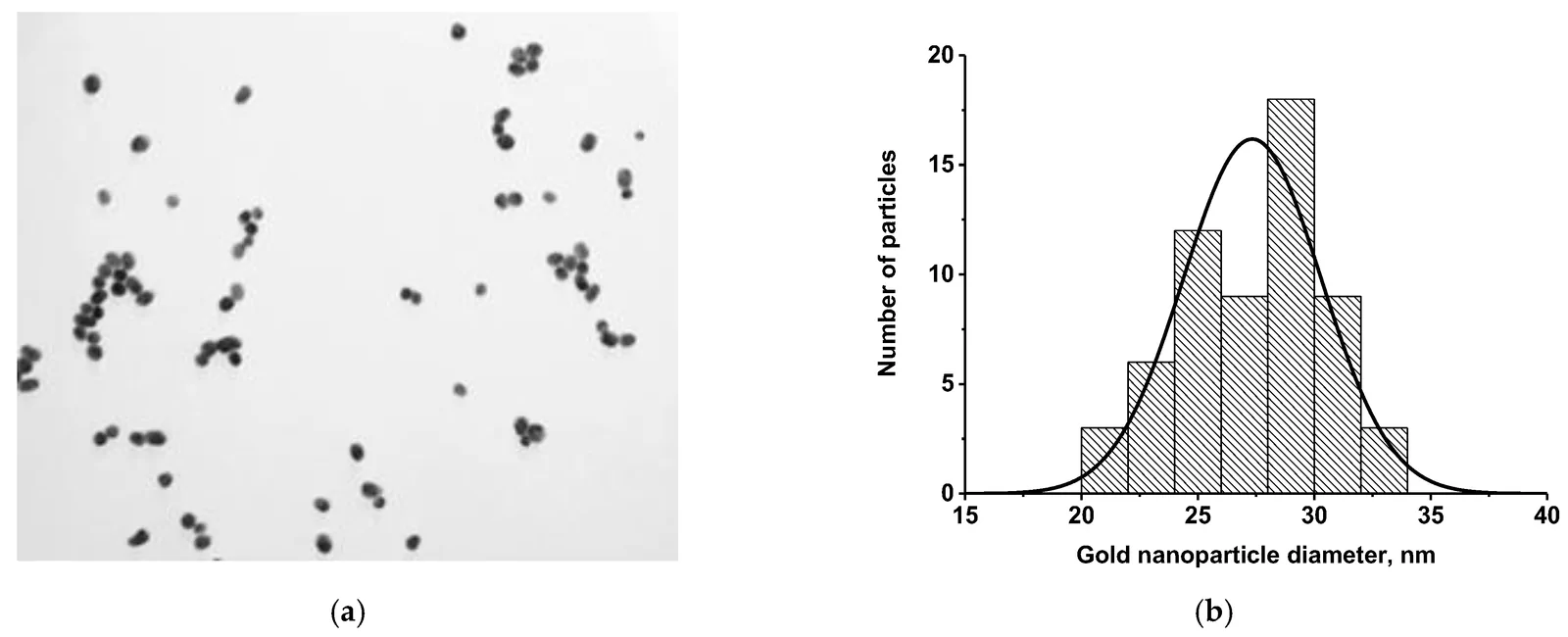
Characterization of AuNPs synthesized for use as the LFIA colorimetric label. (**a**) Transmission electron microscopy (TEM) image; (**b**) corresponding histogram of nanoparticle diameter distribution.

To develop an LFIA for MAB and to comparatively evaluate the performance of LFIA with two types of nanoparticles, AuNPs and Au@CeO_2_ NPs were synthesized. Spherical AuNPs were prepared by the reduction of HAuCl_4_ with sodium citrate according to the method of Frens [23]. For the development of the indirect LFIA for MAB detection, AuNPs were conjugated with polyclonal GAMI antibodies via physical adsorption. The synthesized nanoparticles exhibited a mean diameter of 27.34 ± 2.96 nm (range: 20.89–32.72 nm, *n* = 58) and an ellipticity coefficient of 1.09 ± 0.05 (Figure 1), which is recommended for LFIA that use this kind of nanoparticles [30]. The optimal concentration of GAMI ensuring AuNP stability (6 μg/mL) was selected according to a procedure established in our previous research [24]. The formation of the GAMI–NP conjugates was verified by dynamic light scattering (DLS) measurements performed using a Zetasizer Nano ZS 90 instrument (Malvern, UK).

Au@CeO_2_ nanoparticles were prepared using a seeded-overgrowth strategy, employing Au seeds and precipitating CeO_2_ at alkaline pH. Based on the developments of Piella et al. [25], Ce^3+^ precipitation was performed in the presence of high concentrations of sodium citrate. Citrate anions complex Ce^3+^, slowing the reaction and ensuring high colloidal stability by coating the nanoparticle surface with negatively charged groups.

**Figure 2:**
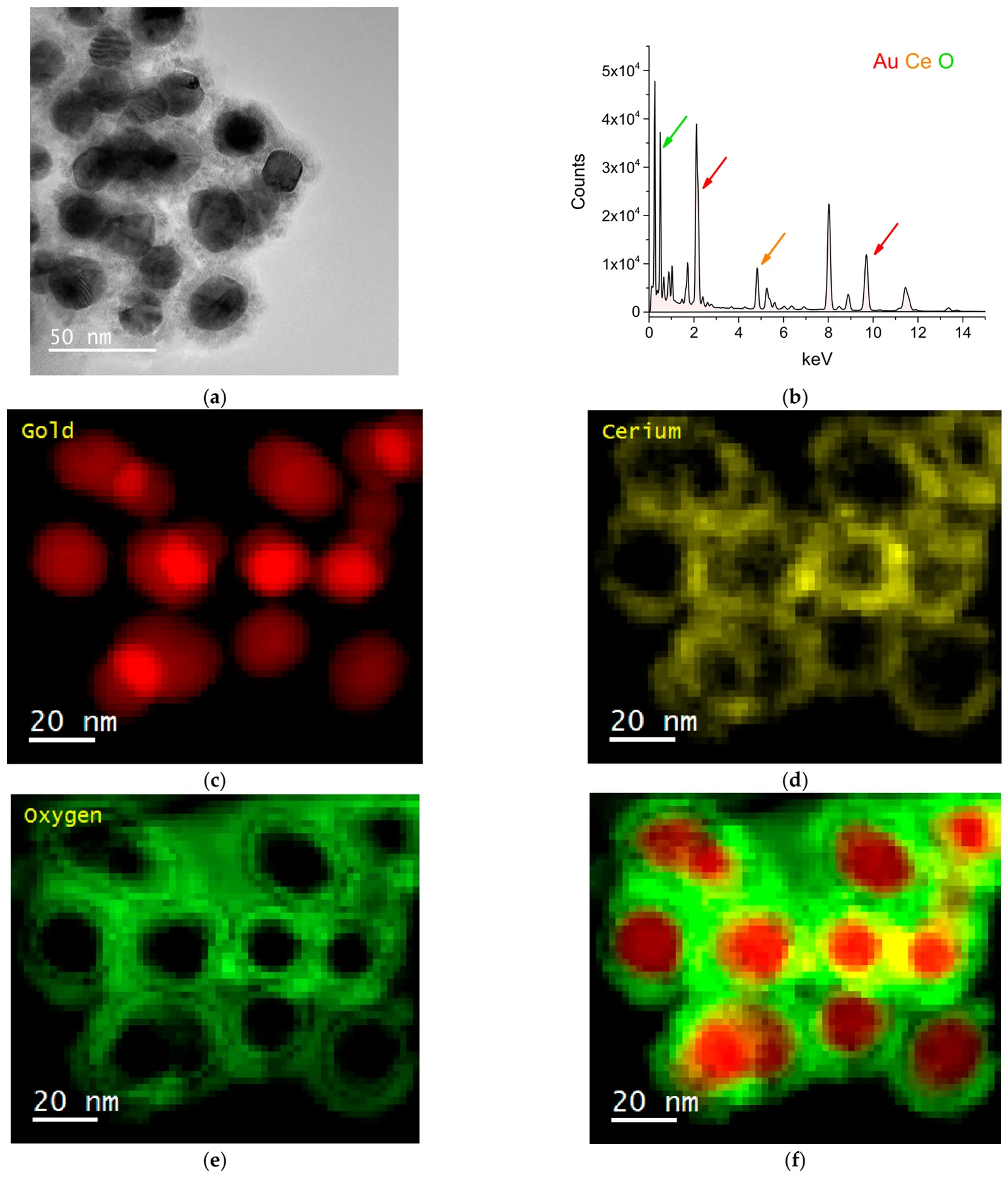
TEM image of Au@CeO_2_ NPs obtained as the LFIA label (**a**), EDX results (**b**), and spatial elemental distribution studied using EELS (**c**–**f**).

TEM results (Figure 2a) confirmed the formation of core@shell-like structures, with Au cores surrounded by a clearly distinguishable shell of lower electron density. The size of the nanoparticles increased to 33.1 ± 2.8 nm compared to the seed AuNPs (16.5 ± 1.4 nm). While EDX results (Figure 2b) confirm the presence of Au (Mα = 2.12 keV; Lα = 9.71 keV), Ce (Lα = 4.84 keV), and oxygen (Kα = 0.52 keV), they do not explicitly confirm the core@shell structure. Spatial elemental distribution was further studied using EELS (Figure 2c–f). These results confirm the formation of core@shell structures, with Au cores surrounded by a Ce-containing shell (Figure 2c,d). The overlapping distribution of Ce and oxygen signals (Figure 2d,e) indicates the formation of a CeO_2_ shell. Kinetic parameters of the peroxidase-mimicking reaction for Au@CeO_2_ were established for both substrates (Figure S2). The apparent K_M_ values were 930 ± 70 mM for H_2_O_2_ and 0.09 ± 0.15 mM for TMB. Under the standard assay conditions, 1.8 M H_2_O_2_ and 0.2 mg/mL TMB (≈0.83 mM) were used. To obtain conjugates of Au@CeO_2_ NPs with anti-species antibodies, GAMI was used at a concentration of 20 μg/mL. Successful antibody adsorption onto the particle surface was confirmed by UV–Vis spectroscopy (Figure S3) and dynamic light scattering. DLS measurements revealed an increase in the average particle diameter from 44 nm for Au@CeO_2_ to 52 nm for GAMI–Au@CeO_2_.

### 3.2. LFIA Format for MAB Detection

We have implemented a heterological format of the competitive immunoassay, where immunogen and coating antigen were synthesized using different kinds of haptens. (Namely, the coating antigen used was prepared by conjugation of a salbutamol derivative—see Section 2.3 and [21]). The heterological approach is known in the literature as an efficient one for modulation of assay specificity [31,32,33,34]. In our case, limited cross-reactivity with salbutamol in solution was accompanied by efficient binding to its derivative immobilized on the membrane surface, which differs in the accessibility and charge of its atoms. On the other hand, the initial screening of the monoclonal antibody against mabuterol demonstrated its suitability for sensitive competitive detection.

**Figure 3:**
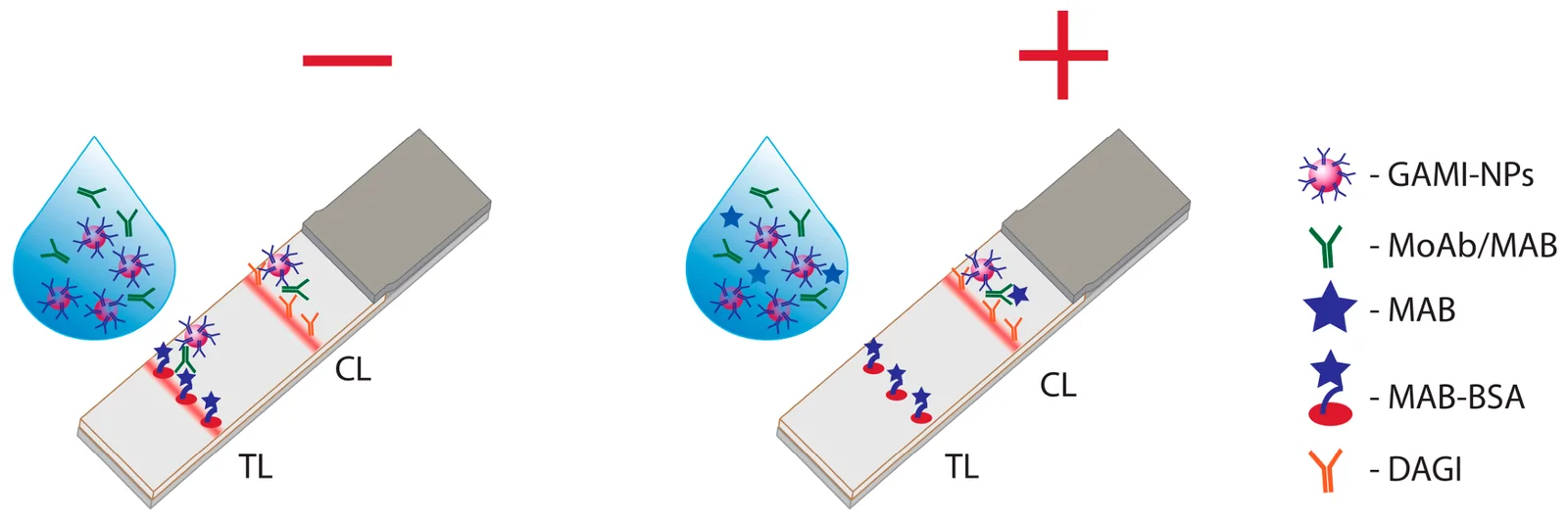
Scheme of the realized LFIA for MAB.

The coating antigen conjugate was immobilized in the TL of the test strip, while DAGI were adsorbed in the CL. Free MAB present in the sample and the immobilized coating antigen conjugate in the TL competitively interacted with the antibodies, forming specific complexes. The label was introduced into the analytical system via a conjugate of nanoparticles with anti-species antibodies. This assay format allows for a significant reduction in the concentration of specific reagents, namely the specific antibodies and the carrier protein conjugate of the target analyte, thereby increasing detection sensitivity (Figure 3).

The test strip was incubated with a mixture containing the sample, specific antibodies, and the GAMI-AuNPs conjugate. In the absence of MAB in the tested sample, a complex consisting of the coating antigen, specific antibodies, and the GAMI-AuNPs conjugate was formed in the TL, resulting in its coloration. When MAB was present in the sample, it blocked the binding sites of the specific antibodies, preventing their interaction with the coating antigen immobilized in the TL. In this case, no coloration was observed in the TL. Regardless of the sample composition, the excess GAMI-AuNPs conjugate interacted with DAGI, producing a CL coloration.

### 3.3. Development of an AuNP-Based Lateral Flow Immunoassay for MAB Detection

The goal of LFIA optimization was to select conditions ensuring maximum assay sensitivity. In competitive analysis, this is achieved by reducing the concentration of immunoreagents involved in the specific recognition reaction, while maintaining sufficient intensity of the formed colored complexes for visual assessment. To demonstrate the analytical feasibility of this and the LFIAs presented below, we have considered their IC_10_, IC_20_, and IC_80_ values to specify the working range and instrumental detection limits based on the widely used practice of LFIAs characterization [35,36,37].

To achieve maximum detectability of MAB, the following parameters were optimized: the working concentration of the coating antigen immobilized on the TL, the concentration of specific antibodies in the reaction mixture, and the type of working buffer. A CNPC12 nitrocellulose membrane with a pore size of 15 µm was selected as the working membrane based on its demonstrated suitability for analyses involving meat matrices [38,39]. In the present study, shortened test strips lacking a sample pad were employed. This configuration reduced the required reaction-mixture volume by a factor of two and decreased the assay time from 15 to 10 min.

Reducing the concentration of the immobilized coating antigen in the test line (TL) from 1 to 0.5 mg/mL maintained clear, uniform coloration of the test zones and provided an opportunity for further optimization of LFIA detectability by reducing the concentration of specific MonoAb. As shown in Table 1, the minimum LOD was achieved using specific antibodies at a concentration of 1.24 µg/mL. However, under these conditions, TL coloration was very weak, with the analytical signal intensity decreasing 4.3-fold compared to the intensity obtained with the maximum MonoAb concentration (6.2 µg/mL). Using MonoAb at concentrations of 3.1 and 1.55 µg/mL yielded identical LOD values for MAB (1.55 ng/mL). However, at a MonoAb concentration of 3.1 µg/mL, the TL coloration intensity was 1.6-fold higher, which justified the selection of this MonoAb concentration for subsequent experiments. DAGI were adsorbed onto the control line (CL) at a concentration of 0.25 mg/mL, which provided an optimal balance of TL and CL coloration intensities during assay performance.

The results presented above were obtained using PBST as the working buffer. It was demonstrated that replacing PBST with 0.01 M MES (pH 7.7) or 0.01 M HEPES (pH 7.4), containing Triton X-100 or Tween 20 as detergents at concentrations of 0.05% or 1%, resulted in a significant decrease in TL coloration intensity or complete absence of coloration. These results confirm that the use of 0.01 M MES (pH 7.7) and 0.01 M HEPES (pH 7.4) in the LFIA for MAB detection is not suitable.

**Figure 4:**
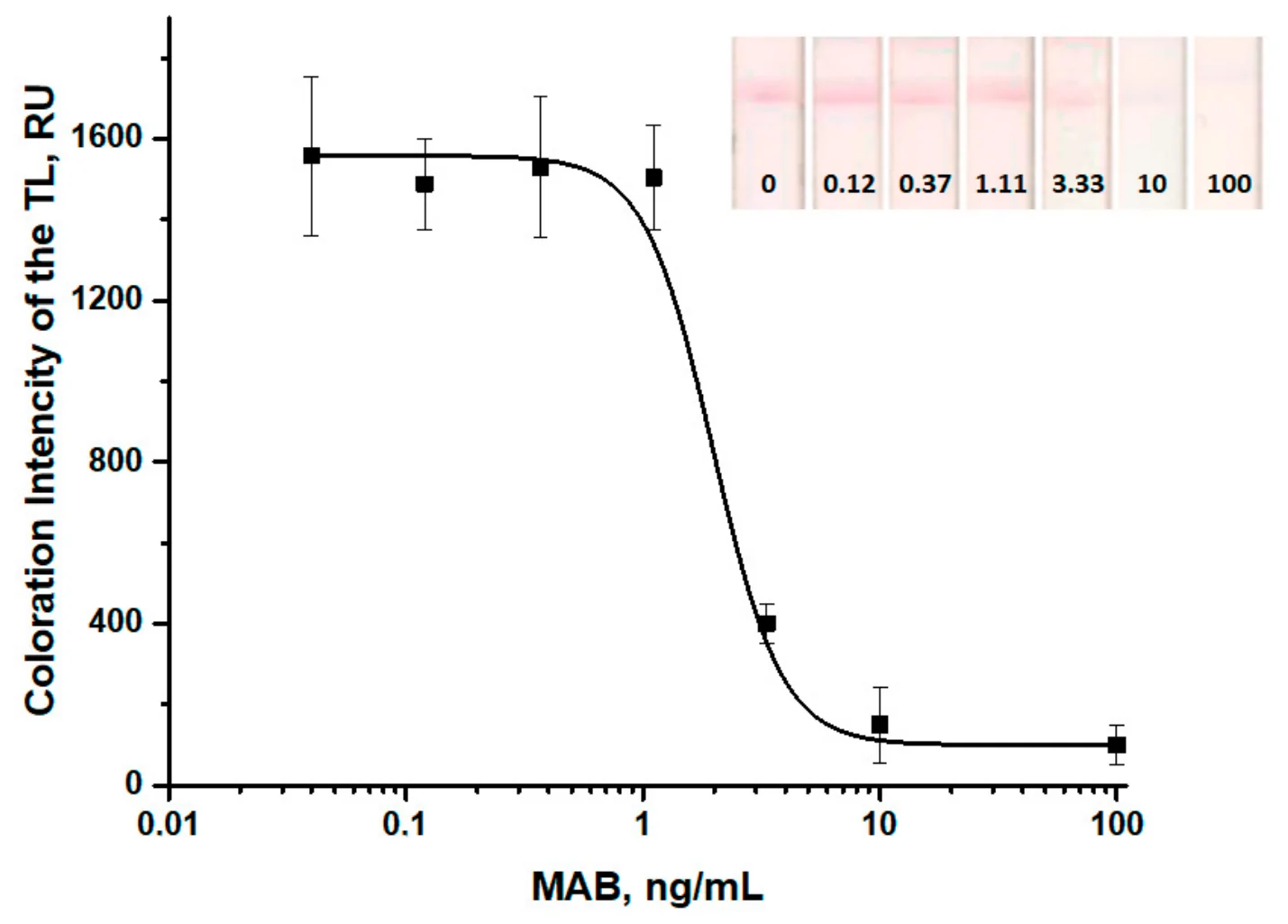
Calibration curve for the LFIA of MAB with AuNPs and images of the corresponding test strips. The concentrations of MAB are as follows: 0 (1), 0.12 (2), 0.37 (3), 1.11 (4), 3.33 (5), 10 (6), and 100 (7) ng/mL (quantity of repeated experiments, *n* = 3).

The optimal combination of high TL coloration intensity and maximum detectability was achieved when using DAGI and coating antigen at concentrations of 0.25 and 0.5 mg/mL, respectively. For the specific MonoAb, the optimal concentration was determined to be 3.1 μg/mL, while the GAMI–AuNP conjugate provided the best performance at OD_520_ = 10.0. Under these conditions, the LFIA calibration curve yielded an instrumental MAB LOD of 1.55 ± 0.16 ng/mL and a detectable working concentration interval of 1.73–3.6 ng/mL. The visual LOD was 10 ng/mL (Figure 4). The total assay time was 10 min.

Cross-reactivity was evaluated using other beta-agonists, including clenbuterol, salbutamol, ractopamine, orciprenaline, terbutaline, and fenoterol. The maximum concentration tested for each analog was 100 ng/mL. The developed test system exhibited 17% cross-reactivity with clenbuterol, whereas cross-reactivity with the other β-agonists was below 0.01% (Figure S4). Given the structural similarity between mabuterol and mapenterol, we expect cross-reactivity with it, but we do not have data for specific consideration of this compound. Furthermore, veterinary drugs belonging to other classes (ampicillin, chloramphenicol, streptomycin, tetracycline) did not inhibit coloration, even at concentrations as high as 1 mg/mL.

### 3.4. Development of an Au@CeO_2_- NPs Based Lateral Flow Immunoassay for MAB Detection

The assay with Au@CeO_2_ was re-optimized to achieve the best analytical performance. As in the AuNP-based LFIA, DAGI and coating antigen were immobilized in the test zones at concentrations of 0.25 and 0.5 mg/mL, respectively.

The use of the Au@CeO_2_ NP conjugate as a catalytic label introduced an additional step into the analysis, i.e., incubation of the test strip with a substrate. A TMB-based substrate mixture was employed [40]. Dextran sulfate is included in the substrate solution to form an insoluble TMB precipitate. The polyanion dextran sulfate captures cationic TMB oxidation products, preventing diffusion of the oxidized product across the test strip and improving signal localization, band sharpness, and reproducibility [41,42]. A high concentration of H_2_O_2_ (1.8 M) has a dual role. First, it matches the high *K_M_* of the nanozyme for H_2_O_2_ (Figure S2a), ensuring a high reaction rate and strong signal amplification. Second, such a high concentration inhibits or inactivates endogenous matrix peroxidases, reducing non-specific substrate oxidation and background.

To evaluate the peroxidase-like properties of the Au@CeO_2_ NPs, the dependence of TL coloration intensity on the interaction time of Au@CeO_2_ NPs with the peroxidase substrate in the presence of hydrogen peroxide was studied over an interval of 2–15 min (Figure S5).

The formed TL and CL were reddish-pink due to the intrinsic color of the Au@CeO_2_ NPs. Upon application of the substrate mixture to the test strip, a gradual increase in the coloration intensity of the test zones was observed. During the first 3 min of incubation with the substrate mixture, the TL coloration intensity increased twofold. Further increase in the substrate incubation time resulted in only a slight enhancement of test zone coloration and the development of background staining. Therefore, the results of the catalytic step were assessed 3 min after substrate mixture application. This approach led to a marked increase in TL coloration and improved the overall analytical performance of the assay.

In summary, the test strips were incubated for 10 min with the reaction mixture containing the sample, specific MonoAb, and the GAMI-Au@CeO_2_ NP conjugate. Subsequently, the strips were placed horizontally, and 5 µL of the TMB substrate mixture was applied to the membrane between the TL and the CL. This volume was sufficient to uniformly cover the TL, absorb the substrate mixture, and form additional coloring. After a 3 min incubation with the substrate mixture, the test strips were scanned, and the coloration intensity of the TLs was quantified.

**Figure 5:**
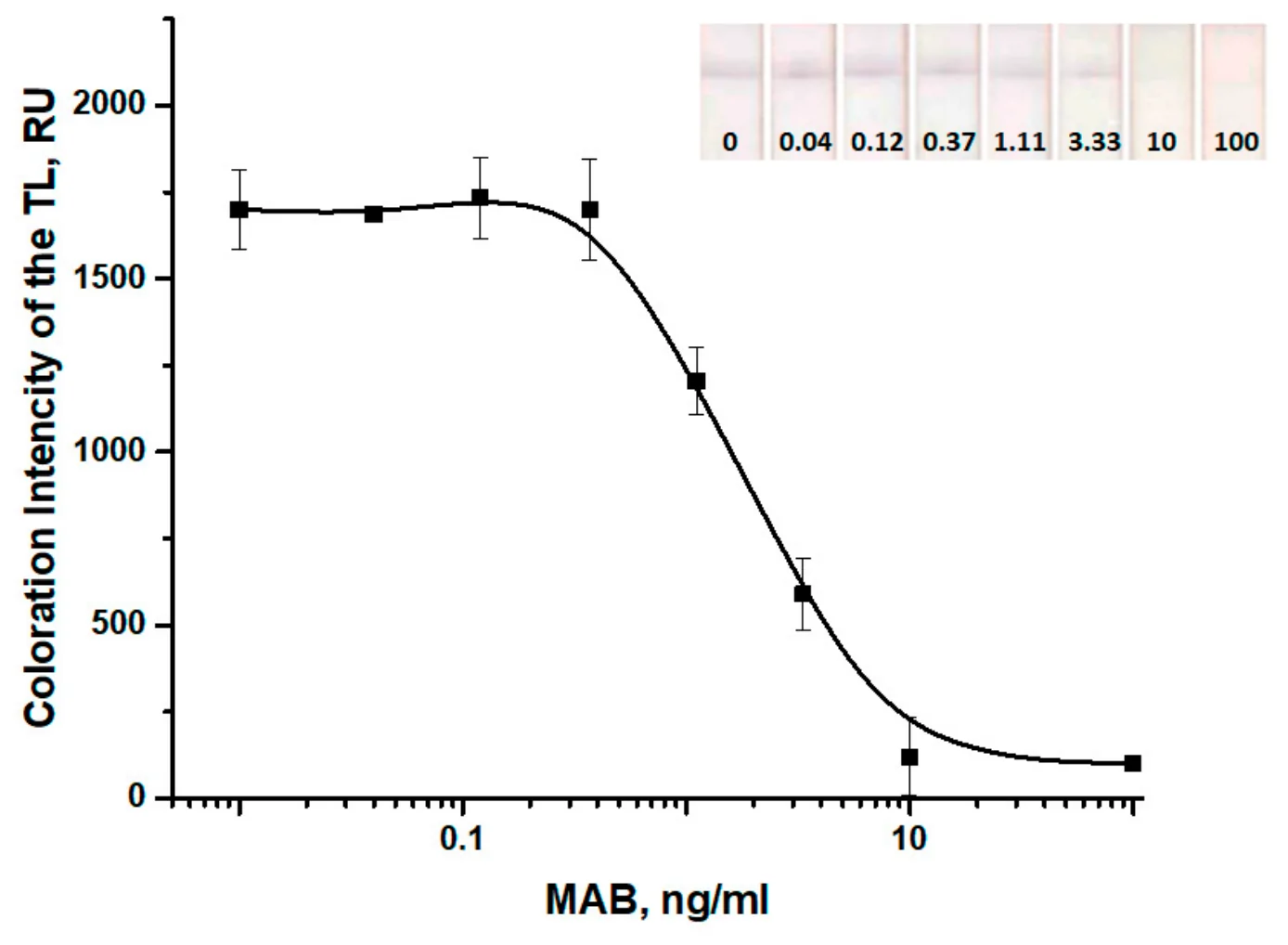
Calibration curve for the LFIA of MAB with Au@CeO_2_ NPs (one-step) and images of the corresponding test strips. The concentrations of MAB are as follows: 0 (1), 0.04 (2), 0.12 (3), 0.37 (4), 1.11 (5), 3.33 (6), 10 (7), and 100 (8) ng/mL (*n* = 3).

The incorporation of an additional substrate mixing step into the procedure increased the signal intensity, facilitating a reduction in the concentration of the specific MonoAb to 1.55 µg/mL. The calibration curve obtained under these conditions demonstrated an instrumental LOD for MAB of 0.6 ± 0.07 ng/mL, with a detectable working concentration range of 0.93–3.7 ng/mL (Figure 5). The visual LOD was 10 ng/mL, while the complete analytical procedure required 13 min.

**Figure 6:**
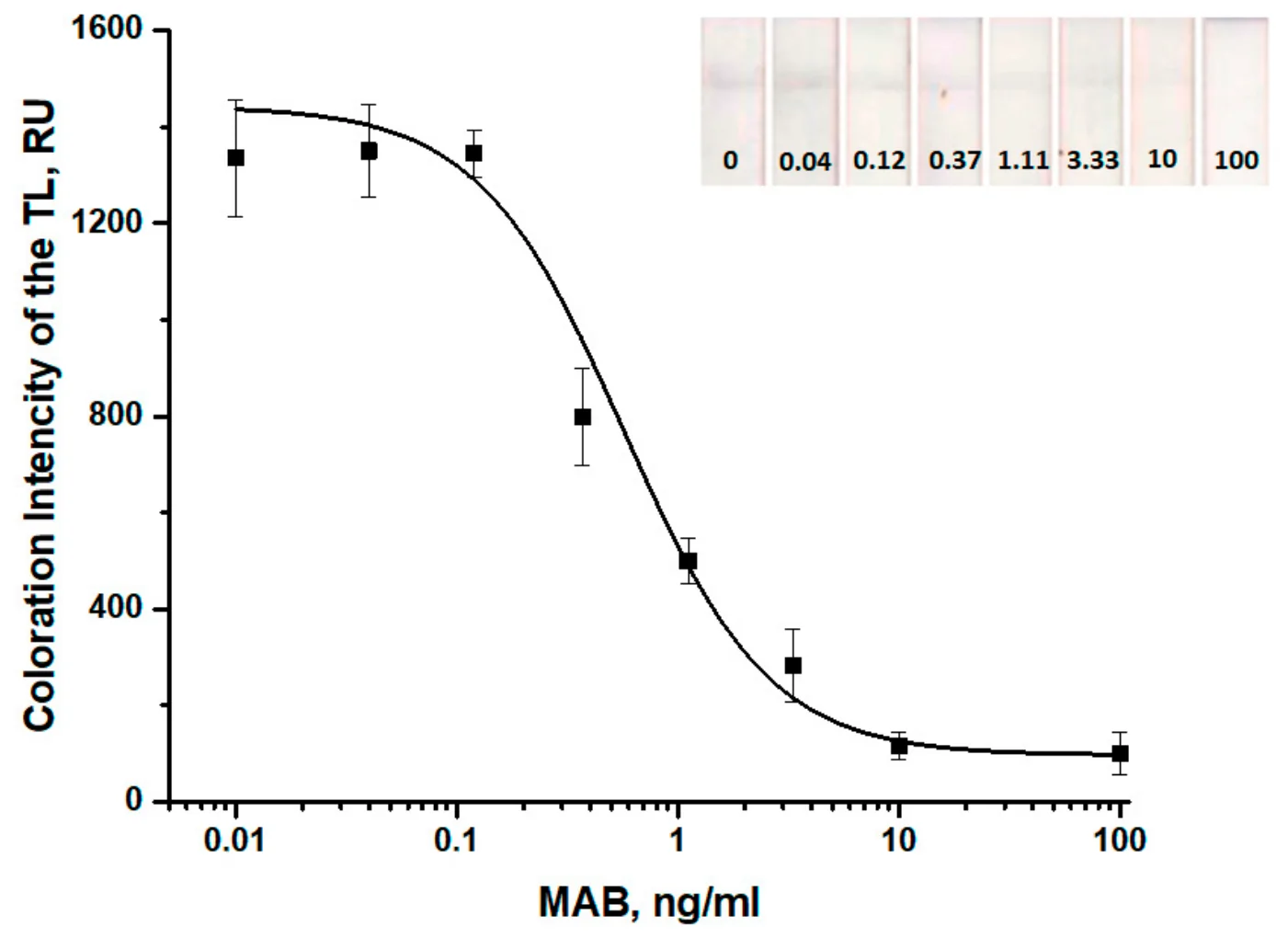
Calibration curve for the LFIA of MAB with Au@CeO_2_ NPs (two-step) and images of the corresponding test strips. The concentrations of MAB are: 0 (1), 0.04 (2), 0.12 (3), 0.37 (4), 1.11 (5), 3.33 (6), 10 (7), and 100 (8) ng/mL (*n* = 3).

Since complex multicomponent metal labels often exhibit nonspecific adsorption on porous membranes [43,44], a two-step assay format was tested. In the first step, test strips were incubated for 5 min with a mixture of the sample and specific antibodies. In the second step, the test strips were transferred for 10 min into a working buffer solution containing the GAMI-Au@CeO_2_ NP conjugate. This scheme allowed additional washing of the test strip with working buffer, facilitating the migration of the GAMI-Au@ CeO_2_ NP conjugate along the membrane during the second assay step. After a 3 min incubation with the TMB substrate mixture, the coloration intensity of the TLs was detected. The calibration curve for the two-step MAB assay, obtained using specific MonoAb at a concentration of 3.1 μg/mL and the GAMI-Au@CeO_2_ NP conjugate corresponding to OD_520_ = 12.0, is shown in Figure 6. Under the optimized conditions, the calibration curve yielded an instrumental LOD for MAB of 0.26 ± 0.04 ng/mL, with a working range of detectable concentrations from 0.32 to 1.39 ng/mL. The visual LOD was determined to be 10 ng/mL. Note that the assay demonstrated an expanded working range of detectable concentrations and a shift to lower MAB content, whereas visual LOD did not differ from AuNPs-based LFIA. The relative standard deviation (RSD) of the measured brightness of the colored band in the TL did not exceed 15.0%. The total assay time was 18 min.

The developed test system demonstrated 21% cross-reactivity with clenbuterol, but cross-reactivity with other beta-agonists, including salbutamol, ractopamine, orciprenaline, terbutaline, and fenoterol, was less than 0.01%.

It was demonstrated that storing the test systems for two months did not result in significant changes in their analytical performance (IC_10_ = 0.26 ± 0.04 ng/mL initially and 0.32 ± 0.03 ng/mL after two months).

The proposed analytical method, like other implementations of nanozyme enhancement in LFIA, involves an additional step after the formation of nanoparticle-labeled complexes in the test zone of the working membrane—the transformation of the chromogenic substrate. Accordingly, the inclusion of an additional reagent in the analytical kit and the increased analysis time should be recognized as limitations of the new analytical method. However, it is based on a widely used commercial chromogen, and the relative increase in total testing time is small. A transition to substrates pre-applied and dried on additional carriers could be considered as a task for further improvement of this approach.

In conclusion, the optimization of the LFIA with catalytic amplification based on Au@CeO_2_ NPs improved the analytical performance of instrumental MAB detection while maintaining the same visual LOD, demonstrating enhanced possibility of colorimetric readout. Specifically, an approximately 2.6-fold decrease in the limit of detection of MAB for the single-step immunochromatographic assay and six-fold for the two-step assay was achieved compared to the AuNP-based LFIA.

### 3.5. Testing of Spiked Extracts

The developed signal-enhanced LFIA method was applied to detect MAB in extracts from real meat samples. Raw meat samples (beef, chicken, pork) purchased from local organic food stores and certified as toxicant-free products were used as real samples for the preparation of extracts. Therefore, no alternative analytical methods were employed to independently confirm the absence of contaminants in the samples.

To study the effect of the sample matrix on the detectability of the developed Au@CeO_2_ NP-based LFIA, the most effective sample preparation techniques were selected. Raw meat samples (beef) were homogenized and processed according to strategies based on: (1) simple buffer extraction [26], (2) extraction with ultrasonic treatment [27], and (3) extraction with an organic solvent followed by evaporation of the obtained extract [28]. Various concentrations of MAB were added to the resulting extracts, which were then used for testing with the developed Au@CeO_2_ NP-based LFIA (two-step).

**Figure 7:**
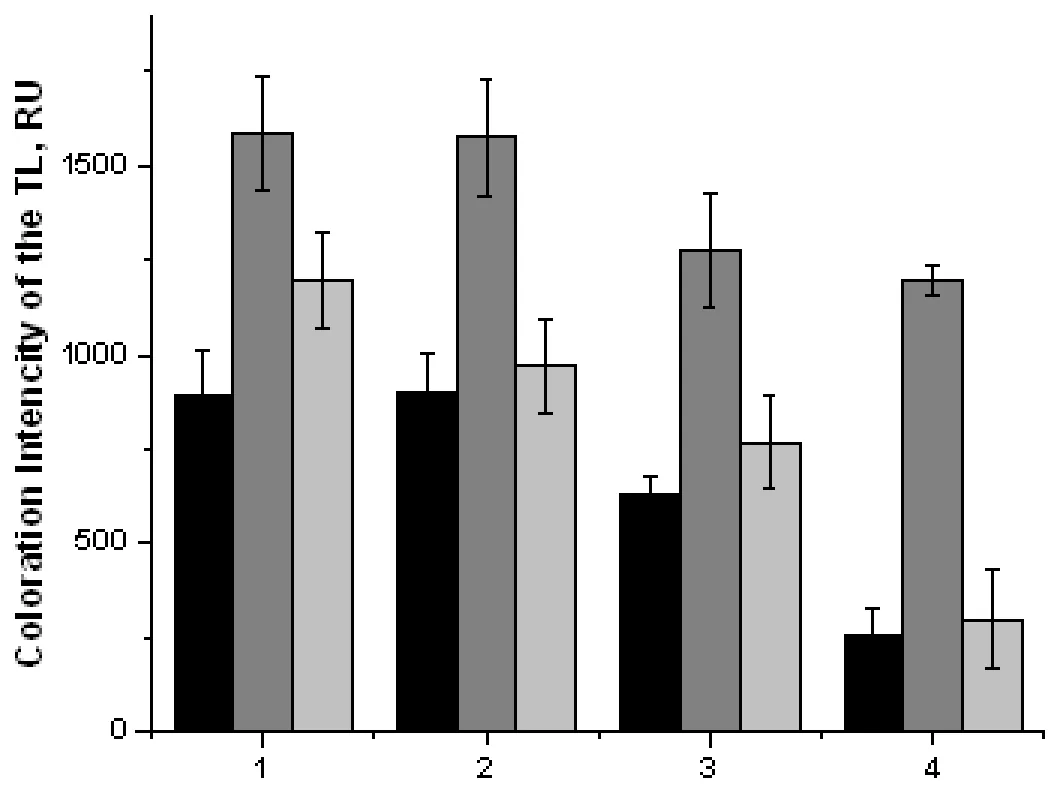
The intensity of TL staining at the concentration of MAB 0 (1), 0.37 (2), 1.11 (3), 3.33 (4) ng/mL in samples treated by three protocols: simple buffer extraction (black column), extraction with ultrasonic treatment (dark gray column), and extraction with an organic solvent followed by evaporation of the obtained extract (light gray column) (*n* = 3).

A substantial decrease in assay detectability was observed, particularly with ultrasonic treatment, which induced maximum disruption of muscle tissue cell integrity. This reduction in assay detectability likely stems from the concomitant destruction of matrix cells and the consequent release of endogenous colored components from samples (e.g., hemoglobin). These components contribute to additional coloration of the test zones (Figure 7). The closest agreement with the MAB calibration curve in PBST was attained using sample preparation method (3), which included extraction, solvent evaporation, and redispersion of the residue in working buffer. This extraction protocol ensures maximum removal of interfering matrix components, which is crucial for the effective performance of the Au@CeO_2_ NP-based system. In this case, the limit of detection for MAB was determined to be 0.26 ng/mL.

During pretreatment, meat samples were incubated with a high concentration of an organic solvent: 2 g of minced meat + 6 mL of acetonitrile. Subsequent aqueous dilutions of this extract, with the addition of the substrate solution used for nanozyme catalysis, did not produce reliably detectable changes in its optical density. This indicates complete inactivation of endogenous peroxidases by this pretreatment.

In the developed assay, we used high H_2_O_2_ concentrations (1.8 M) in the substrate solution both to inactivate endogenous peroxidases and to achieve strong nanozyme-mediated signal amplification.

The efficiency of MAB detection in the presence of components of meat (beef, chicken, pork) extracts using sample preparation method (3) was evaluated at two MAB concentrations selected from the working range of the assay (0.5 and 1.0 ng/mL). The results demonstrate that the developed Au@CeO_2_ NP-based LFIA enables the detection of 80–139% of MAB in spiked meat extracts (Table 2). Although different calibration curves were used for different kinds of meat sources, some detection rates differed significantly from 100% and are therefore rated as relatively acceptable (Figure S6).

The minimal detected content of mabuterol by the developed enhanced test system calculated per weight unit of initial food matrix (for complete extraction 0.26 ng/mL ÷ 1.33 g/mL ≈ 0.2 µg/kg) corresponds to the Codex Alimentarius MRLs for different beta-agonists ranging from 10 μg/kg for ractopamine to 0.2 μg/kg for clenbuterol. The visual cutoff threshold is 7.5 µg/kg (10 ng/mL ≈ 7.5 µg/kg). There are no specifically defined individual MRLs for mabuterol.

Table 3 compares the analytical characteristics of the developed two-step Au@CeO_2_ nanoparticle-based LFIA with those of previously reported methods. The developed system exhibits analytical performance comparable to that of existing LFIAs for MAB and has demonstrated good performance in the analysis of extracts from various types of meat. Although the ELISA (Figure S1) is more sensitive than the LFIA developed in this study, it requires additional analytical equipment, which limits its practical applicability.

## 4. Conclusions

A lateral flow immunoassay was developed for the specific detection of the beta-agonist mabuterol. Substituting conventional gold nanoparticles with bimetallic Au@CeO_2_ nanostructures—which exhibit intrinsic peroxidase-like catalytic activity—improved the limit of detection 2.6-fold for the single-step assay and sixfold for the two-step assay, for which an instrumental LOD of 0.26 ng/mL was achieved. The assessment of matrix effects using beef, pork, and poultry extracts confirmed the practical applicability of the method. The proposed indirect competitive format, leveraging Au@CeO_2_ as catalytic labels, demonstrates broad applicability for the ultrasensitive detection of low-molecular-weight toxicants in food safety monitoring. This approach not only amplifies signal transduction via nanozyme-mediated chromogenic reactions but also offers an alternative to enzyme-labeled conjugates, thereby expanding the analytical toolbox for rapid surveillance.

## Figures and Tables

**Table 1:** Instrumental LODs and TL coloration intensities at different concentrations of MonoAb.

No	Coating Antigen, mg/mL	MonoAb, µg/mL	Instrumental LOD, ng/mL	TL Coloration Intensity, RU
1	0.5	6.2	2.24	3160
2 *	0.5	3.1	1.55	1645
3	0.5	1.55	1.55	1025
4	0.5	1.24	1.14	730

* Finally chosen LFIA conditions.

**Table 2:** Results of the MAB determination in meat extract (sample preparation method (3)) with the developed lateral flow test system (*n* = 3).

Meat Sample	Added Concentration, ng/mL	LFIA, ng/mL	DR, %
Beef	0.5	0.40 ± 0.07	80 ± 14
	1.0	1.18 ± 0.20	118 ± 20
Pork	0.5	0.63 ± 0.08	126 ± 16
	1.0	1.25 ± 0.15	125 ± 15
Chicken	0.5	0.69 ± 0.11	139 ± 20
	1.0	1.34 ± 0.20	134 ± 20

**Table 3:** Comparison of the developed and the published LFIAs for mabuterol.

Techniques/Labels	Limit of Detection	Cross-Reacting Compounds	Real Samples	Reference
AuNPs	Cutoff 10 ng/mL	Mapenterol	Pig urine	[22]
Fluorescent carboxy mod-ified latex nanoparticles	IC_50_ = 0.1 ng/mL	Clenbuterol, brombuterol, cimaterol, cimbuterol, bromchlorbuterol, banbuterol	Pork	[26]
AuNPs	IC_50_ = 1.34 ng/mL	Salbutamol, clenbuterol, brombuterol, clenpenterol, carbuterol, cimbuterol, mapenterol, pirbuterol, terbutaline, cimaterol and clenproperol	Swine urine, lamb samples	[45]
AuNPs	IC_10_ = 1.55 ng/mL Cutoff = 10 ng/mL	Clenbuterol	Meat samples	This work
Au@CeO_2_	IC_10_ = 0.26 ng/mL IC_50_ = 0.56 ng/mL Cutoff = 10 ng/mL	Clenbuterol	Meat samples	This work

## Data Availability

The original contributions presented in this study are included in the article/Supplementary Material. Further inquiries can be directed to the corresponding author.

## Supplementary Materials

The following supporting information can be downloaded at: https://www.mdpi.com/article/10.3390/foods15203732/s1, Figure S1: Calibration curve of MAB detection in the ELISA (*n* = 3); Figure S2: Lineweaver–Burk plots for Au@CeO_2_-catalyzed TMB peroxidase-like oxidation. (a) for H_2_O_2_. (b) for TMB; Figure S3: UV-Vis spectroscopy of Au@CeO_2_ NPs with anti-species antibodies; Figure S4: The cross-reactivity of the developed AuNP-Based test system; Figure S5: The dependence of TL coloration intensity on the interaction time of Au@CeO_2_ NPs with the peroxidase substrate in the presence of hydrogen peroxide; Figure S6: Images of test strips obtained after MAB detection using Au@CeO_2_ NPs (two-step method) in the presence of meat components: beef (a), pork (b), and chicken (c). Sample preparation method (3) was used, and MAB concentrations were selected from the assay’s working range: (1) 10, (2) 3.33, (3) 1.11, (4) 0.37, and (5) 0.12 ng/mL.
