# Supplementary material for: Sensitive Lateral Flow Immunoassay of Beta-Agonist Mabuterol in Meat Samples Using Au@CeO2 Nanoparticles as a Catalytic Signal-Enhancing Label
Source: Foods. 2026 Oct 20;15(20):3732. doi: 10.3390/foods15203732 (PMC13704101; doi:10.3390/foods15203732)
Supplement: Supplementary file 1 [file foods-15-03732-s001.zip › foods-4581737-supplementary.pdf]

Supplementary

Sensitive Lateral Flow Immunoassay of Beta-Agonist Mabuterol in Meat Samples Using Au@CeO_2 Nanoparticles as a Catalytic Signal-Enhancing Label

Elena A. Zvereva [1], Vasily G. Panferov [1], Polina S. Lukina [1], Olga D. Hendrickson [1], Xinxin Xu [2], Chuanlai Xu [2], Boris B. Dzantiev [1] and Anatoly V. Zherdev [1]*

[1] A.N. Bach Institute of Biochemistry, Research Center of Biotechnology of the Russian Academy of Sciences, Leninsky Prospect 33, 119071 Moscow, Russia; zverevaea@yandex.ru (E.A.Z.); vaspanferov@gmail.com (V.G.P.); polinalukina2@gmail.com (P.S.L.); odhendrick@gmail.com (O.D.H.); dzantiev@inbi.ras.ru (B.B.D.)

[2] State Key Lab of Food Science and Technology, School of Food Science and Technology, Jiangnan University, Wuxi 214122, China; xxx89826@163.com (X.X.); xcl@jiangnan.edu.cn (C.X.)

* Correspondence: zherdev@inbi.ras.ru; Tel.: +7-495-9542804

Method S1. Two-step LFIA format of MAB

For the two-step AuNP-based LFIA format, 50 μL of the sample was initially mixed with 1 μL of MonoAb (3.1 μg/mL). The test strip was vertically immersed in this mixture and incubated for 5 min. The strip was then transferred to 50 μL of PBST containing 1 μL of the GAMI–AuNP conjugate (OD520 = 10.0), followed by a further 10 min incubation. Staining was performed similarly to the one-step analysis.

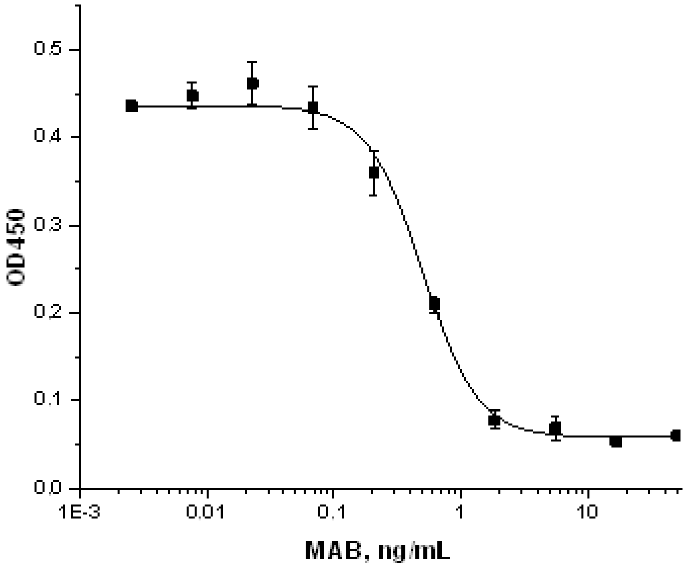

Figure S1. Calibration curve of MAB detection in the ELISA (n = 3).

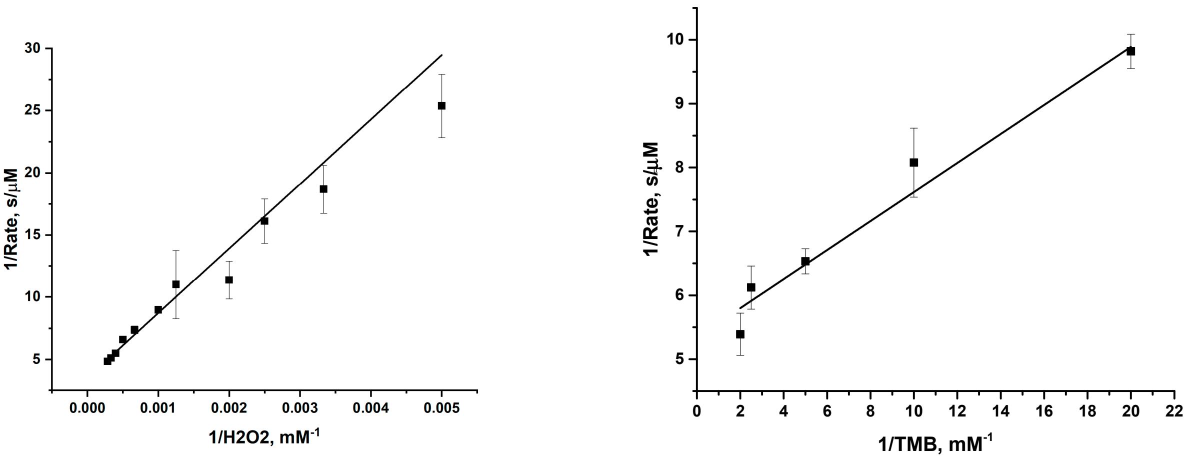

(a) **(b)**

Figure S2. Lineweaver–Burk plots for Au@CeO_2-catalyzed TMB peroxidase-like oxidation. (a) for H_2O_2. (b) for TMB.

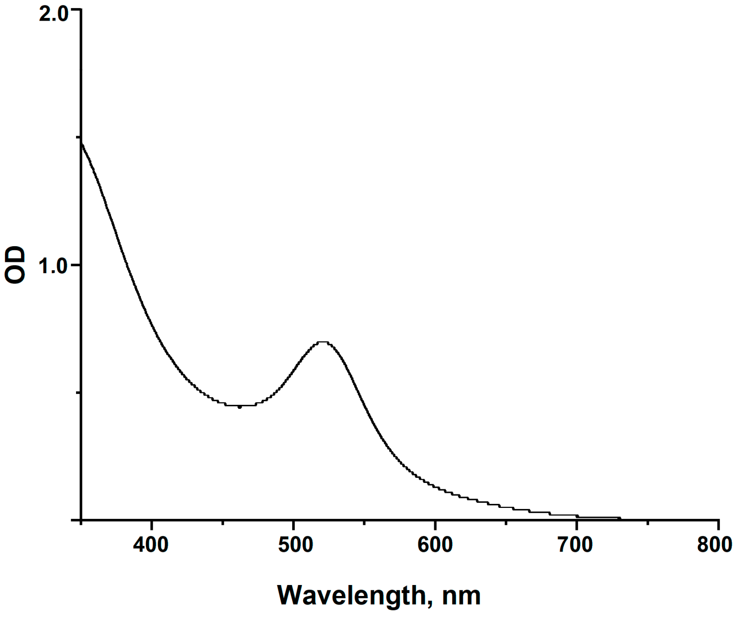

Figure S3. UV-Vis spectroscopy of Au@CeO_2 NPs with anti-species antibodies.

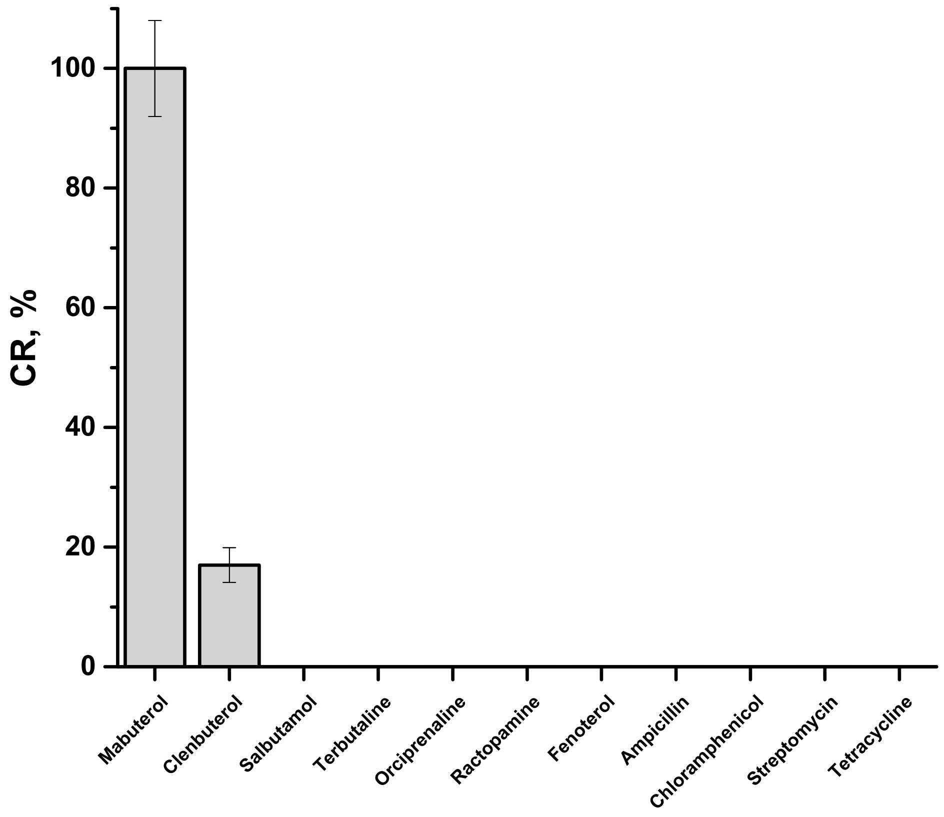

Figure S4. The cross-reactivity of the developed AuNP-Based test system.

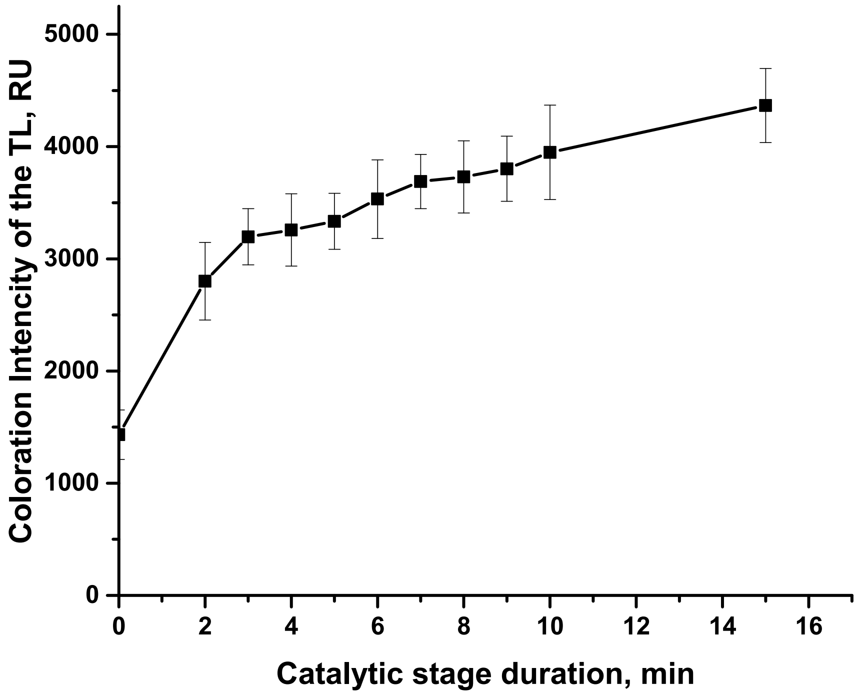

Figure S5. The dependence of TL coloration intensity on the interaction time of Au@CeO_2 NPs with the peroxidase substrate in the presence of hydrogen peroxide.

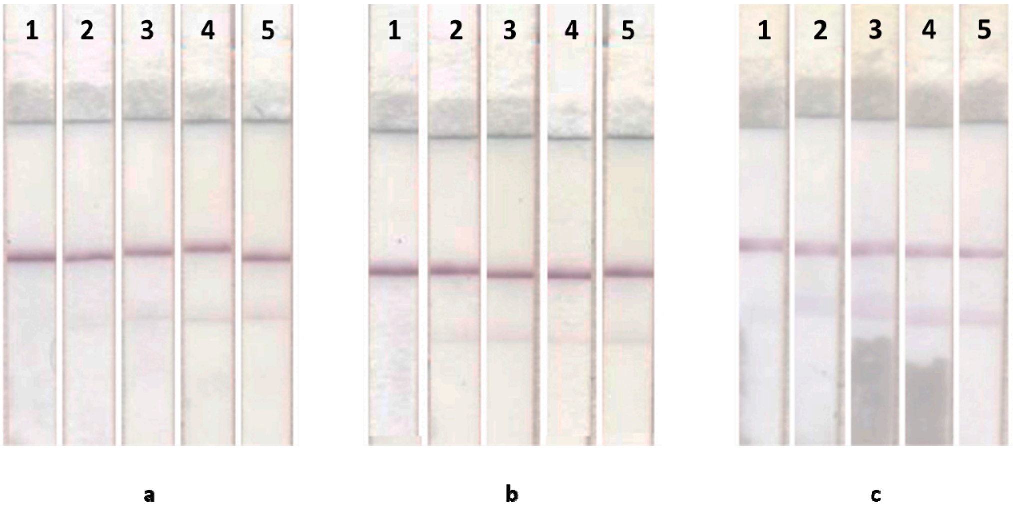

Figure S6. Images of test strips obtained after MAB detection using Au@CeO_2 NPs (two-step method) in the presence of meat components: beef (a), pork (b), and chicken (c). Sample preparation method (3) was used, and MAB concentrations were selected from the assay's working range: (1) 10, (2) 3.33, (3) 1.11, (4) 0.37, and (5) 0.12 ng/mL.
